# P-2162. Is complete avoidance to anti-anerobic therapy possible for empiric fever and neutropenia therapy?

**DOI:** 10.1093/ofid/ofaf695.2325

**Published:** 2026-01-11

**Authors:** Swarn V Arya, Mirae Baichoo, Amandeep Singh, Ravi Singh, Pamela Susman, Marcel van den Brink, Boglarka Gyurkocza, Jonathan Peled, Susan K Seo

**Affiliations:** University of North Carolina Chapel Hill, Durham, NC; Memorial Sloan Kettering Cancer Center, New York, New York; Memorial Sloan Kettering Cancer Center, New York, New York; Memorial Sloan Kettering Cancer Center, New York, New York; Memorial Sloan Kettering Cancer Center, New York, New York; City of Hope National Medical Center, Duarte, California; Fred Hutchinson Cancer Center, Seattle, Washington; Memorial Sloan Kettering Cancer Center, New York, New York; Memorial Sloan Kettering, New York, NY

## Abstract

**Background:**

Use of anti-anaerobic antibiotics has been associated with disruptions of gut microbiota composition and adverse outcomes, including acute graft-vs-host disease in allogeneic hematopoietic cell transplant (allo-HCT) patients (pts). To test the hypothesis that sparing gut anaerobes would confer clinical benefit, we randomized pts undergoing allo-HCT to different regimens for treatment of fever and neutropenia (FN) (NCT03078010). Here we analyzed antibiotic utilization patterns in the trial to evaluate the feasibility of complete avoidance of anti-anaerobic agents.

**Figure:**
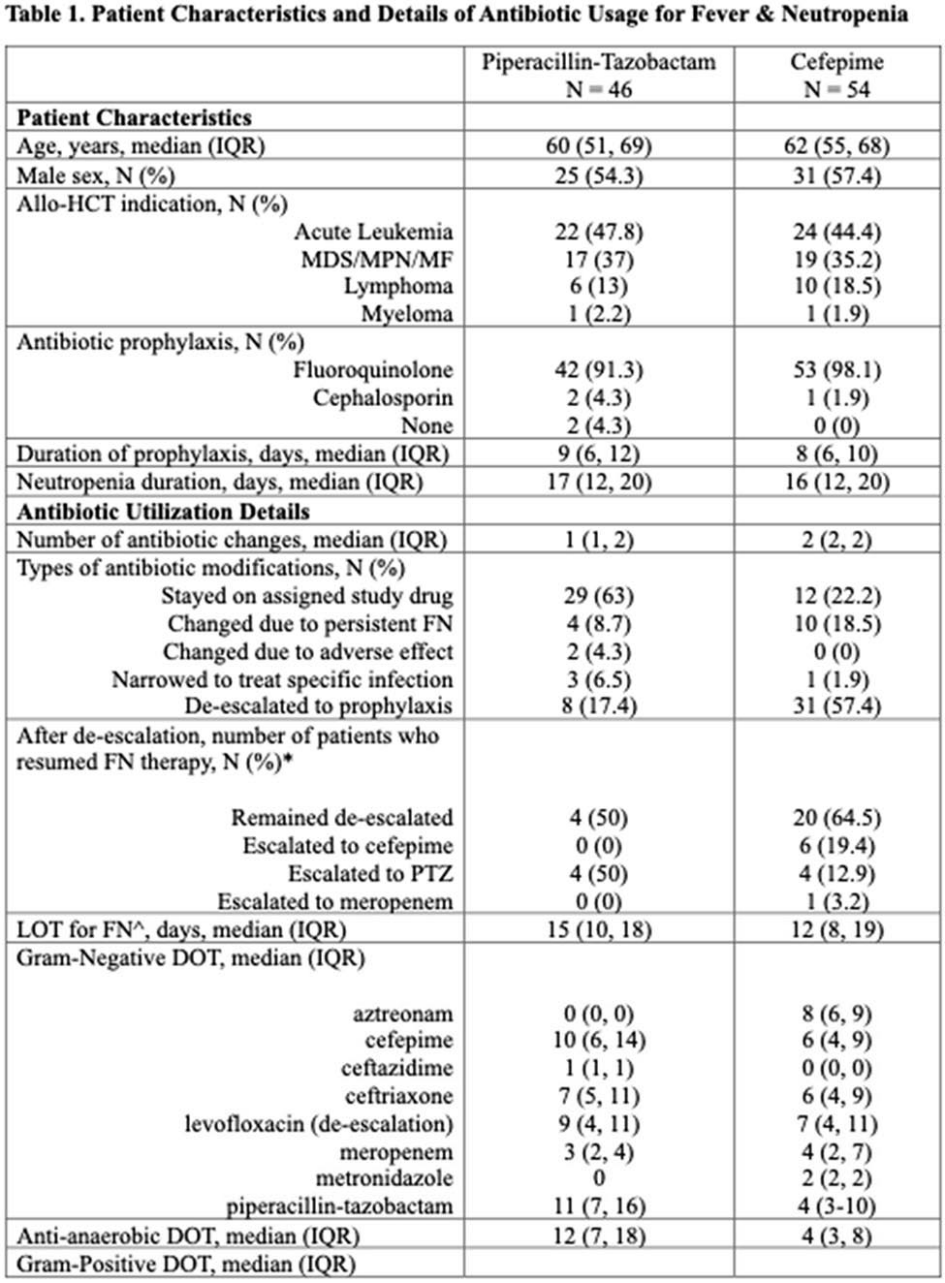

**Methods:**

Pts were randomized to the institutional standard piperacillin-tazobactam (PTZ) or cefepime (CPM) for initial FN. Subsequent antibiotic changes for FN were left to provider discretion. Pts on the CPM arm could be de-escalated to aztreonam (or to a quinolone later in the trial) if clinically stable and afebrile x 72 hours, whereas no formal de-escalation was built into the PTZ arm. The primary outcome was a microbiome endpoint, analysis of which is ongoing. Here, we analyzed anti-anaerobic days of therapy (DOT), which was the sum of duration of days exposed to anti-anaerobic drugs. In contrast, length of therapy (LOT) was analyzed as the duration of days exposed to antibiotics, irrespective of the number of drugs.

**Results:**

There were 54 and 46 evaluable pts in the CPM and PTZ arms, respectively (Table 1). The majority (29/46, 63%) in the PTZ arm remained on PTZ until engraftment. In contrast, more antibiotic modifications were seen in the CPM arm, including 10 (18.5%) switched to PTZ or meropenem for persistent FN, 1 (1.9%) narrowed to ceftriaxone for targeted infection treatment, and 31 (57.4%) with de-escalation. The remaining 12 (22.2%) stayed on CPM until neutrophil recovery. The median (interquartile range, IQR) anti-anaerobic DOT was 12 days (range 7, 18) and 4 days (range 3, 8) for PTZ and CPM arms, respectively.

**Conclusion:**

Antibiotic modifications for FN therapy were common in the CPM arm. Complete avoidance to anti-anaerobic drugs was not possible in the CPM arm, but anaerobic duration of treatment was shorter in the CPM than the PTZ arm. The ability to de-escalate likely mitigated the duration of exposure to anti-anaerobic agents.

**Disclosures:**

Marcel van den Brink, MD, PhD, Ceramedix: Honoraria|Da Volterra: Honoraria|DKMS: Board Member|Garuda: Honoraria|GSK: Honoraria|Juno: IP Licensing|Lygenesis: Honoraria|Nektar Therapeutics: Honoraria|Notch Therapuetics: Honoraria|Pluto Therapeutics: Honoraria|Rheos: Honoraria|Seres: Grant/Research Support|Seres: Honoraria|Seres: IP Licensing|Seres: Stocks/Bonds (Public Company)|Smart Immune: Board Member|Thymofox: Honoraria|Thymofox: Stocks/Bonds (Private Company)|Vor Biopharma: Honoraria|Wolters Kluwer: Royalties Jonathan Peled, MD, PhD, Canaccord Genuity, Inc: Advisor/Consultant|Crestone Inc: Advisor/Consultant|CSL Behring: Advisor/Consultant|DaVolterra: Advisor/Consultant|MaaT Pharma: Advisor/Consultant|Memorial Sloan Kettering Cancer Center: MSKCC Cancer Center Core Grant NCI P30 CA008748|NIH: NHLBI NIH Award K08HL143189|Probiotics Plus Research: Advisor/Consultant|Probiotics Plus Research: Stocks/Bonds (Private Company)|Prodigy Biosciences: Advisor/Consultant|Prodigy Biosciences: Stocks/Bonds (Private Company)|RA Capital: Advisor/Consultant|Seres Therapeutics: Grant/Research Support

